# Common Membranous Sore Throat

**Published:** 1876-04

**Authors:** 


COMMON MEMBRANOUS SORE-THROAT.

It would be improper to conclude these lectures on diphtheria without alluding to a non-specific diphtheric sore-throat, which is often confounded with diphtheria. The local manifestations are similar to those of diphtheria, but their mode of origin is different. Under general subjective symptoms of ordinary catarrhal sore-throat, small vesicles form upon the palate, tonsils, or pharynx, the contents of which become turbid. These vesicles either subside in two or three days, without leaving traces of their presence, or they rupture, leaving small ulcers, which soon become covered with a plastic exudation which spreads and coalesces with other patches which have commenced in the same manner at other portions of the surface.

The prognosis is favorable, the affection subsiding spontaneously in a week or ten days ; though it is occasionally fatal in children, from extension of the pellicle into the air-passages.

The treatment consists in the administration of tonics constitutionally, and the local use of alum, borax, or mild astringents when required.

In the presence of epidemic diphtheria, it would be very difficult to distinguish a case of common membranous sore-throat ; and in case of doubt the better plan would be to treat the case for diphtheria, as unnecessary activity would not be productive of injury. But when these cases get well, as they almost always do, we must avoid recommending for diphtheria some inefficient remedy, during the use of which a case of common membranous sore-throat has gotten well spontaneously. Much of the success attributed to some of the remedies highly lauded in diphtheria has been due to their employment in unrecognized cases of common membranous sore-throat, by no means an unfrequent affection, in large cities, at all seasons.—*Medical Record.*

